# Molecular Diagnosis of SARS-CoV-2: Assessing and Interpreting Nucleic Acid and Antigen Tests

**DOI:** 10.20411/pai.v6i1.422

**Published:** 2021-07-19

**Authors:** Peter A. Zimmerman, Christopher L. King, Mahmoud Ghannoum, Robert A. Bonomo, Gary W. Procop

**Affiliations:** 1 Center for Global Health and Diseases, Case Western Reserve University, Cleveland, Ohio; 2 Center for Medical Mycology and Integrated Microbiome Core, Case Western Reserve University and University Hospitals Cleveland Medical Center, Cleveland, Ohio; 3 Department of Medicine, Case Western Reserve University School of Medicine, Cleveland, Ohio; Louis Stokes Cleveland Department of Veterans Affairs Medical Center, Cleveland, Ohio; Departments of Pharmacology, Molecular Biology and Microbiology, Biochemistry, and Proteomics and Bioinformatics, Case Western Reserve University School of Medicine, Cleveland, Ohio; and the CWRU-Cleveland VAMC Center for Antimicrobial Resistance and Epidemiology (Case VA CARES) Cleveland, Ohio; 4 Cleveland Clinic, Cleveland, Ohio

## Abstract

In this review, we summarize the current status of nucleic acid and antigen testing required for diagnosing SARS-CoV-2 infection and COVID-19 disease. Nucleic acid amplification (NAAT) and antigen-detection (Ag) tests occupy a critically important frontline of defense against SARS-CoV-2 in clinical and public health settings. In early stages of this outbreak, we observed that identifying the causative agent of a new illness of unknown origin was greatly accelerated by characterizing the nucleic acid signature of the novel coronavirus. Results from nucleic acid sequencing led to the development of highly sensitive RT-PCR testing for use in clinical settings and to informing best practices for patient care, and in public health settings to the development of strategies for protecting populations. As the current COVID-19 pandemic has evolved, we have seen how NAAT performance has been used to guide and optimize specimen collection, inform patient triage decisions, reveal unexpected clinical symptoms, clarify risks of transmission within patient care facilities, and guide appropriate treatment strategies. For public health settings during the earliest stages of the pandemic, NAATs served as the only tool available for studying the epidemiology of this new disease by identifying infected individuals, studying transmission patterns, modeling population impacts, and enabling disease control organizations and governments to make challenging disease mitigation recommendations to protect the expanding breadth of populations at risk. With time, the nucleic acid signature has provided the information necessary to understand SARS-CoV-2 protein expression for further development of antigen-based point-of-care (POC) diagnostic tests. The advent of massive parallel sequencing (ie, next generation sequencing) has afforded the characterization of this novel pathogen, informed the sequences best adapted for RT-PCR assays, guided vaccine production, and is currently used for tracking and monitoring SARS-CoV-2 variants.

## IDENTIFYING THE CAUSATIVE AGENT

The first publications reporting patients with a novel pneumonia of unknown etiology from Wuhan, Hubei Province, China, appeared in early January 2020 [1, 2], and used bronchoalveolar lavage (BAL) fluid from 10 adult patients experiencing illness to identify and characterize the causative agent. Patient specimens tested negative by the RespiFinderSmart22kit [3]; this strongly suggested a novel pathogen. From the BAL fluid, transmission electron microscopy captured unmistakable images of a coronavirus. Nucleic acid sequencing technologies demonstrated consistent findings of a novel coronavirus and concordance across hospitalized patients experiencing this novel disease [4], showing 79% sequence homology with SARS-CoV-1 (2003) [5] and an 85% sequence homology with a bat SARS-like CoV (2018) [6]. With this basic clinical and virologic information, development of molecular diagnostic strategies was enabled, including nucleic acid amplification technologies (NAAT) and antigen (Ag) detection and antibody (Ab) analysis. In the sections provided below, we discuss the specimens, collection methods, diagnostic strategies, and the interpretation and significance of test positivity in relation to the prevalence of disease and onset of symptoms. This latter point has become the central concern for diagnosis of SARS-Cov-2 infection and associated COVID-19 illness because of the challenges posed to limiting transmission within and outside clinical settings.

During the earliest days of SARS-CoV-2 emergence (December 2019 to January 2020), studies quickly evolved to document transmission characteristics. Observation of asymptomatic infections (infection without symptoms), human-to-human transmission within multiple family clusters, city-to-city spread (national and international), and detection of transmission from both symptomatic and asymptomatic individuals was described by the end of January 2020 [7–9].

Additional early studies in small cohorts of symptomatic patients showed that viral RNA-positive specimens from the upper and lower respiratory tract (URT and LRT) correlated with onset of symptoms (and viable virus) with URT positivity declining more rapidly than LRT positivity [10–15]. Studies conducted in larger populations including a Chinese pediatric population [16], a uniquely quarantined South Korean religious group [17], and in congregate or other close-quarter living conditions [18, 19] provided insight into some of the idiosyncrasies of diagnostic assay results and COVID-19 symptoms. From studies of this nature, the basic reproductive number (R_o_, R-naught - estimated 2° infections from a single 1° infection) was estimated to range from 1.0-6.95 consistent with continuous human-to-human transmission across sustained transmission chains [20–23]. The convergence of SARS-CoV-2 epidemiology and pathogenesis greatly impacted development of front-line diagnostic tests from methods of specimen collection to virus detection and requires further discussion to understand the challenges of interpretation of results.

## DIAGNOSTIC TEST EFFICACY AND IDENTIFICATION OF INFECTED INDIVIDUALS

**Figure 1.**
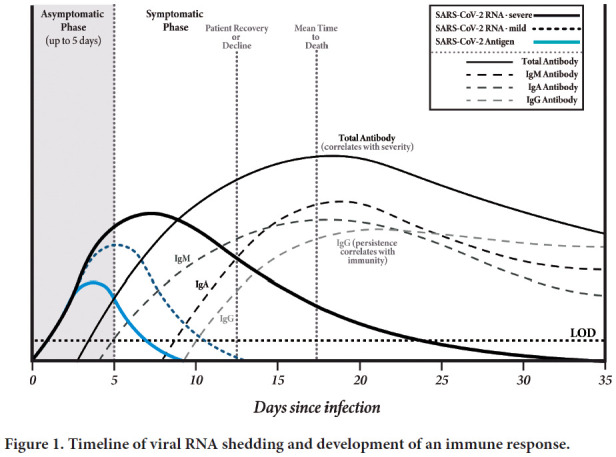
Timeline of viral RNA shedding and development of an immune response.

The central challenge of SARS-CoV-2 infection is the occurrence of asymptomatic infection. The incubation or prodromal phase is characteristic of infectious diseases. Two features of SARS-CoV-2 natural history of infection to disease have driven the COVID-19 pandemic (Figure 1). Firstly, pre-symptomatic people are found to be most highly infectious 1 to 2 days before experiencing symptoms. Secondly, a large percentage of people do not experience symptoms and so are (a) unaware of their infection and never tested, and (b) there is little understanding of **if**, **when**, or **how long** they may be infectious.

Also, as more is learned about the biology of SARS-CoV-2, required components of viral-host cell interaction provide further considerations for optimal specimen collection, observed and unexpected diagnostic outcomes, and association with clinical symptoms.

The 3 most important factors in determining the efficacy of a test are (1) specimen collection/storage/processing, (2) the analytical performance of the test (ie, the sensitivity and specificity, and (3) the nature of the individuals being tested (ie, symptomatic versus asymptomatic). Despite the mundane appearance of this list, it is important to acknowledge that response to the outbreak of this new coronavirus infection required application of diagnostic strategies in a very fluid milieu, simultaneously developing controls for benchmarking the diagnostic assays. As optimization of various diagnostic assays continues, a system for monitoring the resilience of the diagnostic as-says in the face of SARS-CoV-2 evolution has not yet been developed. Given the rapid emergence of COVID-19, it has not been surprising that intense scrutiny has been focused on diagnostic test performance and validity, and that measures of these metrics vary across studies.

The greatest likelihood for detecting SARS-CoV-2 relies on specimen collection from the site of viral replication. This practice has been vetted as part of public health testing for influenza and other respiratory pathogen measures and adapted to the COVID-19 pandemic [24–27]. In this regard, specimen collection and NAAT performance have undergone simultaneous development in becoming the standard for SARS-CoV-2 diagnosis.

Biological materials used for SARS-CoV-2 clinical and research diagnostics have included URT (nasopharyngeal swab, oropharyngeal swab, nasal mid-turbinate swab, anterior nares swab, nasopharyngeal/nasal wash, saliva), LRT (sputum, BAL fluid), and urine, serum and feces. Guidance for these specimen collection procedures are provided by the CDC [27]. In a systematic review of SARS-CoV-2 RNA detection across specimens collected from different anatomical locations, Mallet et al found that RT-PCR test sensitivity varied with timing of specimen collection in proximity to a patient's exposure [28]. Additionally, detection of viral RNA was more successful and for greater duration from LRT than URT sampling, albeit studies performed early in the pandemic were often undertaken in more seriously ill patients. More specifically, virus detection from nasopharyngeal swab was 89% (95% CI 83-93%) when sampled between 0 to 4 days post-symptom onset and 54% (95% CI 47-61%) when sampled between 10 to 14 days post-symptom onset [28]. When comparing detection of viral RNA between specimens collected from patients undergoing both URT and LRT collections, URT sites cleared faster than LRT sites (UTR—median 12 days, 95% CI 8 to 15 days; LRT—median 28 days, 95% CI 20 to not estimable). While virus was detectable in fecal samples for over 45 days, detection varied greatly within individual participants. Additional factors that could contribute to diagnostic test outcome variability include different expression patterns of host proteins known to be receptors for SARS-CoV-2 endothelial cell invasion (reviews on molecular interactions [29–31].

Because of increased ease of collection, significantly reduced patient discomfort, and shortages of nasopharyngeal swabs or personal protective equipment, significant attention was focused on saliva testing as an alternate specimen type for COVID-19 detection [32]. Interest in saliva sampling further increased when researchers from Yale reported a higher viral load in first-morning saliva specimens compared with the traditional nasopharyngeal swab specimen [33]. For context, both Chen et al [34] and Fernández-Pittol et al [35] found saliva to be less sensitive than nasopharyngeal swab (NPS) specimens, while Procop et al found saliva to be an acceptable alternate specimen type to diagnose COVID-19 in the ambulatory setting, with the warning that they reported overall lower median viral load in saliva compared to NPS specimens [36]. Similarly, Pasomsub et al [37] reported a sensitivity and specificity for saliva compared with NPS of 84.2% and 98.9%, respectively, with a 97.5% agreement. Sensitivity of saliva collection is improved with instructional coaching. Kojima et al observed that saliva from the coached group gave more sensitive results than the NPS specimens (90% versus 79%, respectively), but less sensitive in the non-coached group (66%). The investigators reported that several members of the non-coached group failing to elicit a cough (part of their instructions) may have contributed to lower sensitivity results [38].

Despite differences in study outcomes, many have suggested that the testing of saliva could be used to screen asymptomatic, as well as symptomatic individuals with a high degree of efficacy [33, 39]. Yokota et al studied 2 cohorts of asymptomatic individuals. The first cohort had close contact with individuals with confirmed COVID-19, whereas the second cohort required testing for airline travel; together, 1,940 consented individuals were included in this study [39]. Among those in the close-contact group (n=177), SARS-CoV-2 was detected in 44 saliva specimens and 41 NPS specimens. In the asymptomatic travelers (n=1,763), SARS-CoV-2 was detected in 4 saliva and 5 NPS specimens. Their overall analysis reported a sensitivity for saliva and NPS swab testing of 92% (90% CI: 83%-97%) and 85% (90% CI: 77%-93%), respectively, with both collection approaches achieving close to 100% specificity. These studies concluded that saliva collection was straightforward, non-invasive, and minimized the risk to healthcare workers [33, 39].

After appropriate specimen collection, most of the laboratory tests for SARS-CoV-2 employ some type of nucleic acid extraction by one of numerous methods [40–42]. This step removes amplification inhibitors from a concentrated nucleic acid substrate. Some of the rapid RT-PCR and iso-thermal amplification assays do not include a nucleic extraction step, which may slightly decrease the sensitivity of these assays.

## MOLECULAR DIAGNOSTIC TESTS (RT-PCR AND OTHER NAATS)

In general, highly sensitive tests (requires nucleic acid extraction, amplification, and often manual reporting) can be completed within 10-16 hours, meeting a 24-hour turnaround time unless the diagnostic laboratory is backlogged. Some platforms offer highly sensitive assays that are also rapid (eg, SARS-CoV-2 Xpress [Cepheid] [43]). Molecular diagnostic tests include RT-PCR assays, as well as other methods of nucleic acid amplification. A critical first step requires *in silico* and laboratory testing to evaluate oligonucleotide primer and probe hybridization sites prior to committing a SARS-CoV-2 assay into use; target regions of the SARS-CoV-2 genome have included the RdRP, N (N1 and N2), S, E and ORF1ab genes. With primers and probes optimized, tests can then be performed as highly sensitive and moderately sensitive NAATs. Moderately sensitive as-says usually attain a more rapid time-to-result by the exclusion of the nucleic acid extraction step. Approaches that skip this step cannot compete with the extraction/RT-PCR assay with respect to assay sensitivity, as disclosed in the comparative data provided by the FDA (see above) and by numerous independent studies [43, 44]. Extraction-free, rapid tests are best reserved, in the opinion of these authors, for symptomatic individuals who are early in the course of disease and expected to have a higher viral load in their clinical specimens compared to asymptomatic individuals. Of note, FDA Emergency Use Authorization clearance for SARS-CoV-2 assays is focused on testing of symptomatic individuals for the vast majority of assays with this clearance. FDA guidance advises that if a patient is symptomatic and the test result is negative by a moderate sensitivity test, the provider should consider a follow-up with an RT-PCR assay.

Test sensitivity naturally considers the lower limit of detection (LOD), which is the target concentration at which a test will be positive 95% of the time. The target nucleic acid concentration is implied to correspond to the amount of virus in the collected specimen. The LODs of many commonly used SARS-CoV-2 assays have been compared against an FDA reference panel to generate comparative data. [45, 46]. The specificity of the tests addresses the accuracy of the detection. This is largely accomplished in RT-PCR assays by selecting probe hybridization sites that are unique to the microorganisms of interest; selective PCR primers can also influence specificity. For example, it is mandatory that SARS-CoV-2 RT-PCR diagnostic assays do not detect normally circulating coronaviruses (ie, common cold; CoV-229E [α], NA63 [α], OC43 [β], HKU1 [β] [47]), human genes, or genetic elements from other microorganisms that may be present in the specimen materials collected.

More recently, SARS-CoV-2 has been incorporated into multiplex assays that detect other respiratory viruses. Two different classes of multiplex assays have been produced. One of these targets select viral pathogens that are more likely to produce severe disease (ie, SARS-CoV-2 with influenza or SARS-CoV-2 with influenza and respiratory syncytial virus [RSV]). These assays, some of which produce rapid results, will be critically important if SARS-CoV-2 co-circulates with influenza and/or RSV in future respiratory viral seasons. Others have included SARS-CoV-2 with much larger multiplex panels that include the majority of respiratory viruses important in human disease (eg, parainfluenza, adenovirus, etc.). These panels are most commonly used for immuno-compromised patients or children requiring hospitalization. The large multiplex panels are considerably more expensive than individual RT-PCR assays, and are not recommended for routine screening for COVID-19.

The impact of the nature of the patient being tested on the ultimate performance of the test is often overlooked or underappreciated, although its affect is considerable. When assessing test performance, it is also critically important to distinguish whether a test is being used as a *diagnostic test* or a *screening test*. Applied as a *diagnostic test*, there should be a high pre-test probability that the patient is infected. This would most commonly occur because the patient is demonstrating the signs and symptoms of infection. In this instance, the high-performing test will have a high positive predictive value (PPV). This same test with excellent sensitivity and specificity will perform very differently if it is used as a *screening test*, as in testing asymptomatic patients prior to surgery or another medical procedure, who have not had exposure to a person with COVID-19. In this scenario, the PPV decreases significantly, which means that there will be an increased number of false positive results. As an example, the PPV (ie, the likelihood that a positive test result represents actual disease in the patient) of a very good test (ie, 95% sensitivity/95% specificity) diminishes from 95% when there is a prevalence of 50% (eg, in the context of a diagnostic test) to 16% when the prevalence is 1% (eg, in the context of a screening test). To state it otherwise: at a prevalence of 1%, only 16 out of 100 positive results are correct, the remainder (84 out of 100) are wrong (ie, false positives) [48].

## CONSIDERATIONS ON CROSSING THRESHOLD (CTS)

Rapid cycle or real-time PCR is inherently quantitative. Specimens that contain a large amount of target organism become positive early during testing, whereas those with few organisms become positive in later reaction cycles. For example, if a PCR test consists of 45 reaction cycles, then specimens that became positive at cycle 15 would contain significantly more target in the original specimen than a specimen that became positive at cycle 35. When PCR or RT-PCR is used with quantitative standards, these assays can produce highly accurate viral loads, which is done for HIV, CMV, and other viruses. There is a 10-fold difference in target concentration for every 3 PCR cycles. Therefore, the point at which the specimen becomes positive (ie, crosses the threshold or Ct) has been used as an approximate surrogate for the viral load or viral burden in the specimen. Although useful in some situations, there are a number of hazards that must be considered and avoided. Foremost, quantitative standards are not available for SARS-CoV-2, which means a highly accurate determination of the amount of virus present in a specimen cannot be determined. Additionally, specimens from the upper respiratory tract are more heterogeneous, in contrast to a plasma specimen (used in diagnosis of virus populations circulating in the blood). Upper respiratory tract specimens vary from deep nasopharyngeal collection that thoroughly sample respiratory epithelium to sampling of the superficial anterior nares, with specimens varying from thick and viscous to watery. Furthermore, considering the large number of different tests available for which Ct values can be derived, the RT-PCR assays will likely perform with different amplification efficiencies, and are therefore not comparable one to another, without optimized quantitative standards.

Therefore, at best, Ct values at present may be separated into early, mid, and late categories, which correspond to high, medium, and low viral loads, respectively. Although these values have been correlated with outcome in patients with COVID-19 [49–51], the variability of Ct values between different assays and the heterogeneity of specimen collection makes it impractical to attempt to use this metric in any more than a semi-quantitative manner. Crossing threshold (Ct) values have been most effectively used in the experience of the authors to clarify or confirm clinical findings. The RT-PCR results from a patient with acute, symptomatic COVID-19 illness, will usually have an early or mid-range Ct value (ie, a high or medium viral load). Conversely, patients with remote disease who are asymptomatic may still test positive with the highly sensitive RT-PCR assays, but their test results disclose a late Ct or a low viral load.

Crossing threshold values have been very useful in the evaluation of individuals who test positive with asymptomatic screening prior to surgery. Most instances, in our experiences, have disclosed a late Ct value, and the patient has been found to have remote or convalescent disease. In far rarer instances wherein the patient was serendipitously detected in the pre-symptomatic phase, the Ct value was low indicating a higher viral load.

The issue of persistently positive post-infection test results warrants special mention. The residual positivity that is detected by the highly sensitive RT-PCR likely represents the shedding of non-infectious fragments of virus and/or very low levels of virus. Attempts to culture the virus from specimens of this nature have only rarely been successful [52, 53]. Furthermore, although there is epidemiologic evidence that these individuals do not transmit the virus effectively, this finding does not afford the relaxation of mitigation strategies. The residual positivity that occurs poses a significant challenge in obtaining 2 negative tests for back-to-work policies [15]. The CDC, therefore, no longer requires 2 negative tests to return to work, but rather provides a symptoms-based approach [54].

To summarize, while there has been a desire to identify a Ct value after which a patient can be designated as non-infectious, it is not possible at the present time [52]. The absence of absolute quantitation, the heterogeneity of the specimens received, and the differences in the amplification efficiencies of the various SARS-CoV-2 assays all influence the Ct value that is generated.

## SARS-COV-2 GENOME SEQUENCE VARIATION AND ITS IMPACT ON DIAGNOSIS AND DISEASE

A review of essential factors influencing the performance of diagnostic tests must acknowledge the potential for valid and optimally functioning tests to be impacted and/or fail because of sequence variation in the SARS-CoV-2 genome. Since the emergence of SARS-CoV-2, the Global Initiative on Sharing Avian Influenza Data (GISAID), Nextstrain, the National Center for Biotechnology Information (NCBI), and the China National Center for Bioinformation (CNCB) have been aggregating full and partial SARS-CoV-2 genome sequence from infections around the world. These data repositories have been providing the ability to track how the virus is changing over time and space, allowing a basic assessment of the virus' mutation rate and the capacity to monitor priority segments of the viral genome (eg, RT-PCR primer and probe annealing sites, antibody binding sites, receptor binding sites, and viral proteins targeted for vaccine development).

As we have passed the 1-year anniversary of SARS-CoV-2 emergence, recent manuscripts (with over 46,000 sequences [55, 56] and counting) have identified more than 12,700 mutations across the 30,000 bp genome, where the occurrence of non-synonymous compared to synonymous mutations were almost 2-fold greater (NS:S = 1.88) [55]. Phylogenetic analysis has revealed 7 distinct clades shown below using the naming strategy put forth by GISAID in association with gene-specific mutations, *Clade L* (original Wuhan strain; NC_045512.2); *Clade S* (NSP4 [Non-Structural Protein]:S76S, ORF8 [Open Reading Frame]:L84S); *Clade V* (NSP6:L37F, ORF3a:G251V); *Clade G* (S [Spike]:D614G); *Clade GH* (S:D614G, ORF3a:Q57H); *Clade GR* (S:D614G, N [Nucleocapsid]:RG203KR); *Clade GV* (S:D614G, S:A222V), and one additional cluster of numerous low frequency mutations (*Clade O* = other) [56]. Basic research articles that have reported and continue to monitor viral sequence variation examine the geographical distribution and temporal stability of the SARS-CoV-2 clades [57, 58], emergence [59], and phenotypic associations [60, 61] of the spike protein, and preliminary association of disease severity and within-patient viral diversity [62]; the most recent updates on naming strategies have been written by Trevor Bedford and Nextstrain colleagues [63]. Germane to this review are the following observations linked to SARS-CoV-2 genome evolution.

A number of reports have performed assessments of nucleotide sequence variation within the RT-PCR primers and probes creating mismatches and potentially contributing to false-negative outcomes [47, 64–66]. The B.1.1.7 or United Kingdom (UK) variant (now referred to as the Alpha variant), which until recently was the dominant strain in the United States, is associated with S gene target failure (SGTF) in some RT-PCR assays [67]. Similarly, others have described nucleocapsid (N) gene and envelope (E) gene target failures associated with different variants [68, 69]. The hybridization characteristics of the primers and probes of current SARS-CoV-2 RT-PCR assays should be assessed against the rapidly spreading B.1.617.2 or Delta variant, and any subsequent emerging variants to assure assay efficacy. A summary of the information focused on primer and probe sequences has found a higher risk of mismatch (more susceptible to RT-PCR failure) in the N gene, whereas sequence in the E and RNA-dependent RNA polymerase (RdRP) genes have a lower risk of mismatch (less susceptible to RT-PCR failure) [47, 64–66]. As most of the primers and probes used in current diagnostic tests have been observed to hybridize to altered sequence that would result in mismatch, concerns arise regarding potential for false-negative results. The FDA has provided guidance concerning nucleic acid sequence alterations in SARSCoV-2 variants and the impact on diagnostic testing [70].

Emergence of the spike protein D614G substitution in February 2020 has caused significant further investigation [71]. Further diversification of SARS-CoV-2 strains carrying this amino acid change (Strains G, GH, GR, and GV) has received special attention because spike protein 614G strains now predominate throughout most of the world [56, 72]. Of particular interest, 614G (compared to 614D) is observed to be more open to binding the angiotensin-converting enzyme 2 (hACE2), the primary receptor for infection of human endothelial cells and has been associated with higher SARS-CoV-2 viral loads [61].

Because vaccine development commenced very shortly after the SARS-CoV-2 genome was sequenced, these efforts were committed to the sequences available in January 2020. Since this preceded emergence of the D614G sequence variation, vaccine development has been based on the D614 spike protein sequence. Therefore, it is important to determine whether 614G would reduce effectiveness of spike protein recognition by vaccine-induced antibodies. With the whole spike protein gene provided in the Pfizer and Moderna mRNA vaccines, the human immune response and resulting antibodies would be expected to bind to multiple epitopes of the SARS-CoV-2 spike protein. Early studies suggested that the D614G sequence variation is not likely to affect the effectiveness of these vaccines [73]. More recently, studies have shown that continuing emergence of new spike protein variation (K417N, or E484K, or N501Y mutations) is associated with decreased effectiveness of potent neutralizing monoclonal antibodies against recombinant viral strains [74], or predict spike protein mutations that may lead to strains that could escape immune response stimulated by the recently released vaccines [75].

It is critical, therefore, that whole viral genome sequencing is supported on a national level to monitor strain variation, determine the impact on diagnostic tests and viral transmissibility, and assess monoclonal antibody therapy and vaccine efficacy. The CDC maintains an updated website on Variants of Interest, Variants of Concern, and Variants of High Consequence, as well as their proportional distribution across the United States [76].

## ANTIGEN DETECTION TESTS [77]

Antigen detection tests, in general, rely on 2 independent reactions. The first of these is the antigen-antibody interaction. This is the portion of the assay that directly affects the specificity of the reaction. Antibodies directed against particular antigens are raised and harvested from an animal or an immortalized cell line. These antibodies are immobilized on a surface, such as a nitrocellu-lose strip or in the wells of a testing plate. The processed clinical specimen is then allowed to react with the antibody. If the antigen of interest is present, then an antigen-antibody complex will form. The second portion of the reaction is a signal amplification reaction designed to visualize or detect the presence of the antigen-antibody complex. The reaction may be colorimetric or fluorescent and may be detected visually or with instrumentation. This test method has been used in clinical laboratory medicine and physician offices for many years.

The performance of antigen testing in microbiology is well understood as there is abundant literature and years of experience. The principles, advantages, and limitations that have been described for Group A *Streptococcus,* respiratory viral antigen testing, and other antigen tests for infectious agents hold true for antigen tests for COVID-19 tests [78, 79]. Critical to the performance and PPV of these or any tests are the analytic test performance characteristics of the test (ie, sensitivity and specificity) and the population that is being tested.

Antigen tests for respiratory viral pathogens are less sensitive than RT-PCR assays. Unfortunately, the LOD of antigen tests are not included within the current FDA diagnostic assay development guidelines. The FDA should include the LOD of these assays for comparative purposes, as a public service to patients and providers alike. Antigen tests are *most useful* to confirm clinically suspected infections (ie, in a symptomatic patient). The PPV (ie, the likelihood that a positive test result is a true positive) is high when the test is used in this setting. Additional advantages include ease of test performance, a quick time to diagnosis, and a low cost.

When the antigen test is negative on a symptomatic patient (ie, a test result/clinical mismatch), then the patient should be retested with an RT-PCR assay. Occasional false-negative test results are expected to occur due to the limited sensitivity of antigen tests compared with RT-PCR. This recommendation is consistent with CDC guidance, which has stated: “…it may be necessary to confirm a rapid antigen test result with a nucleic acid test, especially if the result of the antigen test is inconsistent with the clinical context” [80], To state it otherwise: If there is a mismatch between the clinical findings and the test result, then an RT-PCR should be performed.

As previously indicated in the comparison between diagnostic test (likely all symptomatic individuals with high viral load) and the screening test (likely more asymptomatic individuals with low-to-no viral load) setting, the likelihood of false positives increases (see example, below). Application of tests outside populations intended for use has contributed to numerous examples of false positive SARS-CoV-2 antigen tests reported in the lay press. Not surprisingly, these occur-rences cause excitement in the general public and uncertainty in the validity of the diagnostic test arsenal.

Additionally, there is a *significant patient safety issue* if these tests are used to triage or cohort patients. As an example, if uninfected patients with false-positive results were placed in close proximity with patients who were truly infected, these false-positive patients would encounter inappropriate risk and increased possibility of becoming infected. If antigen tests are used to screen asymptomatic patients, then positive results should be considered presumptive positives, until confirmed by an RT-PCR test. In an epidemiological / public health context, there is also a danger of artificially inflating infection rates if positive results from asymptomatic patients are reported without RT-PCR confirmation. A comparison of the benefits and limitation of antigen testing and RT-PCR for SARS-CoV-2 is provided (Table).

**Table.** The Benefits and Limitations of Antigen and RT-PCR Testing^1^ for SARS-CoV-2^2^

Parameter	Antigen Testing	RT-PCR	Comments
Sensitivity	Moderate	High	Nucleic acid amplification tests are generally more sensitive than antigen detection tests. Early infection (ie, lower viral loads) would be expected to be missed more frequently with tests with lower sensitivity.
Specificity	High	High	Specificity depends on assay design (eg, the antigen-antibody interaction for antigen tests, and primer/probe selection for RT-PCR, among a variety of other factors).
Cost	Low	Moderate to High	Cost will vary based on platform and the inclusion of nucleic acid extraction step prior to RT-PCR.
Time-to-Result	Fast	Fast-to-Slow	Antigen test results are generally available in 30 minutes or less. There are some isothermal and one RT-PCR platform that offer quick time-to-results (ie, an hour or less), but generally testing with in-laboratory RT-PCR can be completed within a day. Time to result varies significantly by platform, transport time, and specimen backlog.
PPV^3^ in a High Pretest Likelihood Setting^4^	High	High	When there is a high pretest likelihood of infection/disease, antigen tests perform very well, despite a sensitivity that is not as great as RT-PCR.
PPV^3^ in a Low Pretest Likelihood Setting^5^	Low-to-Moderate	Moderate	All tests produce false positive reactions when used in low prevalence settings, but generally antigen detection tests suffer from this to a greater degree than RT-PCR assays.^6^

1. The RT-PCR assays considered here include a nucleic acid extraction prior to amplification and are performed in a laboratory, unless otherwise noted.

2. This comparison assumes well-designed assays that are performed according to the manufacturer's instructions or current Emergency Use Authorization (EUA) guidance.

3. PPV = Positive Predictive Value of the test result (ie, a positive test truly represents the presence of SARS-CoV-2).

4. A high pretest likelihood setting would consist of a patient demonstrating signs and symptoms consistent with COVID-19 and/or testing occurring in the setting of a high prevalence of disease in the community.

5. A low pretest likelihood setting would consist of a patient without the signs and symptoms consistent with COVID-19 (eg, asymptomatic screening) and a low prevalence of disease in the community.

6. The higher rate of false-positive reactions for antigen detection tests is likely because only a single aberrant reaction (ie, an antigen-antibody reaction) is necessary for a false positive test result, whereas for traditional RT-PCR several aberrant reactions (eg, 2 primers and a probe mis-hybridization) must occur to produce an erroneously positive test result. Specimen mis-handling (eg, splashing, mix ups) can occur with any test and are not considered here.

## SPECIMEN POOLING

There has been much interest in pool testing, given the limitations in reagents and other materials required for testing. In pooled testing, aliquots of multiple specimens (eg, 8-10) are combined and tested together rather than being tested individually.

The advantage of this approach is the preservation of testing reagents. For example, if a pool of 10 samples tested negative using a single test-worth of reagents, all of the negative results would have been generated at a 10-fold savings. If a pool of specimens tested positive, the pool would be “deconstructed” and each specimen would be tested individually to determine which specimen or specimens were the cause of the positive reaction. Pooling has been used to detect HIV and HBV in donated blood or in resource limited settings [81, 82], but has not been used in clinical laboratories, until now [83].

When specimens are pooled there is a *dilution effect*. If a specimen is pooled wherein the target analyte is at or near the LOD, then that specimen may be mischaracterized as negative. We studied pooling in asymptomatic patients with COVID-19 and demonstrated an 85% correlation between direct testing and those pooled 10:1, which meets the FDA criteria for pooling [84]. The only specimens missed after pooling were those with low viral loads (ie, late Cts). The impact of a false negative differs depending on the phase of disease (ie, early versus late), immunologic status of the patient, the clinical (eg, admission of a patient into an ICU vs ambulatory testing) or public health circumstances. These and similar factors should be considered to determine which patients are eligible for pooling.

Pooling specimens also requires significant handling and pipetting of potentially infectious clinical specimens with numerous opportunities for human error (eg, mislabeling to contamination events) which would impact quality assurance of a certified clinical laboratory. Additionally, as the prevalence of infections rise, more and more pools will test positive, and necessitate deconstruction of the pools and re-testing of the component specimens. This has a significant impact on both labor and reagents, with a diminishing return on savings.

## CONCLUSION

In this brief summary we present the current state of the art regarding nucleic acid amplification testing and antigen testing. We hasten to add that this is a continually evolving field and that variants are likely to be more common as we apply comprehensive genome sequencing to different isolates. Sequence variability is predictable, as is the possibility of escape variants emerging in the clinic. At this point, efforts to detect COVID-19 by multiple methods should still be pursued. As the genetic landscape becomes more complicated, we will need multiple tools to assist us in the implementation of appropriate diagnosis, the administration of therapy, and the execution of infection control. We remain entirely optimistic that we will find the appropriate testing algorithms and technologies that will help us overcome this global threat.
